# Acceptability of HPV Vaccines and Associations with Perceptions Related to HPV and HPV Vaccines Among Men Who Have Sex with Men in Hong Kong

**DOI:** 10.1371/journal.pone.0057204

**Published:** 2013-02-22

**Authors:** Joseph T. F. Lau, Zixin Wang, Jean H. Kim, Mason Lau, Coco H. Y. Lai, Phoenix K. H. Mo

**Affiliations:** 1 Centre for Health Behaviours Research, The Jockey Club School of Public Health and Primary Care, Faculty of Medicine, The Chinese University of Hong Kong, Hong Kong, China; 2 Centre for Medical Anthropology and Behavioral Health, Sun Yat-sen University, Guangzhou, China; University of Cape Town, South Africa

## Abstract

HPV vaccines are available to men but there are few studies investigating the acceptability of HPV vaccines among men who have sex with men (MSM), a high risk group. We assessed the intention to take up HPV vaccines among MSM in Hong Kong and the associated factors related to cognitions on HPV and HPV vaccines, basing on the Health Belief Model (n = 542). The acceptability of HPV vaccines was 20% (unconditional on efficacies and price), 29.2% (conditional on efficacies and market price), 51.7% (conditional on efficacies and discounted price) and 79.1% (conditional on efficacies and free price). Adjusting for background variables, composite scores of perceived susceptibility, perceived severity, perceived barriers and cue to actions were significantly associated with acceptability of HPV vaccines conditional on specific efficacies and the market price. Acceptability of HPV vaccines was highly price sensitive. Future studies need to use conditional measures. Implementation and translational researches are warranted.

## Background

HPV is highly infectious. It causes not only genital warts as a sexually transmitted disease (STD) but also cervical cancer among females and anal/penile cancer among males [1]. Previous studies conducted in the U.S. have documented high prevalence of HPV in the male general population, ranging from 7.9% [2] to 63.1% [3]. One recent study estimated that the prevalence of genital warts is 0.94% in the Hong Kong male general population, with an estimated incidence of about 300 per 100,000 person-years [4], which was higher than those estimated in the U.S. and in other countries [5].

Recent studies have shown that HPV vaccines could effectively prevent genital warts, penile and anal cancers, and reduced high grade anal lesion (HGAIN) among males [6], [7], [8]. A number of cost-effectiveness studies have been conducted on HPV vaccination. One recent review study pointed out that “Cost-effectiveness studies indicate that in the context of modest female vaccination rates and with the specification of a broad range of disease outcomes (e.g. genital warts, anogenital cancers, and oropharyngeal cancers), male vaccination can be quite cost-effective” [9]. One cost-effectiveness study pointed out that “The most cost-effective scenario would include vaccinating MSM at age 12 before exposure to HPV; however, vaccination occurring up to age 26, with exposure to all vaccine types assumed to be 50%, was still cost-effective” [10]. Another study also concluded that “Vaccinating boys and men age 9–26 against all HPV 6/11/16/18-associated diseases provides substantial public health benefits and is cost-effective at commonly cited thresholds” [11].

The U.S. Center for Diseases Control and Prevention (CDC) recommends men who have sex with men (MSM) of age 26 years or younger to take up HPV vaccines [12], followed by the European Medicines Agency [13], Society of Physicians of Hong Kong and the Family Planning Association of Hong Kong [14]. Although the recommended age range for males is 9–26 years old, doctors can opt to give it to other men “off license” if they wish and some doctors recommend its use to adult men. One study showed that the quadrivalent HPV vaccine significantly reduced high grade anal lesions (HGAIN) recurrence among MSM of 20–70 years old [8]. In addition, two ongoing clinical trials are investigating the efficacies of HPV vaccines targeting males of age >26 years older [15], [16]. Although current programs promoting HPV vaccination only target MSM of age < = 26 years old, it is potentially important to understand acceptability of HPV vaccination among MSM of age >26 as it is possible that new researches may find HPV vaccines efficacious among older males.

HPV and HIV infections are closely related to each other, whilst MSM in general greatly concern about HIV. The prevalence of genital and anal warts was 8.9% and 19.6% respectively among Australian HIV-negative homosexual men [17]. A study showed that the prevalence of genital warts was 13.2% among MSM in China [18]. A 61-city study further reported HIV prevalence among MSM in China to be 4.9% [19], whilst two cohort studies reported high HIV incidences of 4.17 and 5.40 per 100 person-years [20], [21]. Co-infection of HPV and HIV are consequential [22]. One study showed that carcinoma and anal intraepithelial neoplasia were frequent in HIV-positive men [23], whilst another study showed that “all HPV-associated cancers in AIDS patients occurred in statistically significant excess compared with the expected numbers of cancers” [24]. Furthermore, some studies showed that anal HPV infection may be associated with HIV acquisition [25] and that HIV incidences increased significantly with high risk HPV positivity [26]. The potential relationships between HPV and HIV and the concern of HIV among MSM may motivate MSM indirectly to take up HPV vaccination.

Six published studies have investigated acceptability of HPV vaccines among MSM. The prevalence of acceptability ranged from 36% to 86% [27], [28], [29], [30], [31], [32]. Only one of these studies mentioned the price of HPV vaccines when asking the question on acceptability [32]. Significant associated factors included perceived susceptibility and perceived severity of HPV-related diseases, perceived effectiveness, benefits and positive attitudes related to the vaccines, belief that a physician would recommend the vaccines to MSM, anticipated regret for contracting HPV without taking up HPV vaccines and number of sex partners. Acceptability studies are important in designing effective HPV vaccination promotion campaigns. None of the aforementioned studies was conducted in China.

Health promotions basing on health behavioral theories are more likely to be effective than non-theory-based interventions [33]. The Health Belief Model (HBM) specifies that the constructs of perceived susceptibility and perceived severity in contracting HPV, perceived benefits, perceived barriers, cue to action and self-efficacy related to taking up HPV vaccines are determinants of HPV vaccination. It has been applied to explain and to promote HPV vaccination among females [34] and was used in this study to guide variable selection.

We therefore investigated the prevalence of intention to take up HPV vaccination in the next six months among MSM age 18–60 years old in Hong Kong under four scenarios (see Measures) and perceptions related to HPV and HPV vaccines derived from HBM. Associations between acceptability (conditional on efficacies and market rate) and the HPV-related perceptions were also investigated.

## Subjects and Methods

### Study Population and Data Collection

Inclusion criteria were: 1) Hong Kong Chinese men of 18 to 60 years old and 2) having had oral or anal intercourse with at least one man in the last six months. Participants were recruited from some gay venues (bars and saunas) frequently visited by gay people during September 2010 through January 2011. This method has been used in previous studies [35], [36]. Recently, a mapping exercise was conducted by the government and identified 12 gay bars and 16 gay saunas in Hong Kong [37]. All these venues were approached by our research staff. Approval was sought from the owners of six of the gay bars and nine of the gay saunas. The venue-based interviews were administered by some experienced peer interviewers at different time slots during weekdays and weekends. They briefed prospective participants about the study and invited them to join the study. Convenience sampling was performed at the venues. A total of 1100 eligible MSM were invited to join the study; 550 refused to participate; 8 participants did not complete the interview; 542 participants (49.3%) provided written informed consent and completed the anonymous face-to-face interview in a setting with privacy ensured. They were given HK$50 (about US$6) for their time spent (about 20 minutes). Ethics approval was obtained from the ethics committee of the Chinese University of Hong Kong.

### Measures

Participants were asked about their socio-demographic information, sexual orientation, utilization of HIV-related services, history of STD (symptoms and diagnosis), unprotected anal intercourse (UAI) with men and the number of male sex partners with anal intercourse in the last six months (see Table 1). Acceptability was assessed under four scenarios: 1) without mentioning efficacies and price (unconditional), 2) conditional on the efficacies of taking up three shots of HPV vaccines within three months (>90% for prevention of genital warts and 75% for prevention of HPV induced cancers [38]) and a market price of HK$1000 to 2000 (US$ 128 to 256) per shot for three shots, 3) conditional on the aforementioned efficacies and about half of the market price (HK$500 to 1000; US$64 to 128) per shot for three shots, 4) conditional on the aforementioned efficacies and three free shots.

**Table 1:** Frequency distributions of the background variables (N = 542).

	N	%
Background characteristics		
Age group		
18–25	138	25.5
26–30	151	27.9
31–40	195	36.0
41–60	58	10.7
Highest education attained		
Junior high or lower	25	4.7
Senior high	173	31.9
College and above	344	63.5
Marital status		
Single/Divorced or widowed	461	85.1
Cohabiting with men	69	12.7
Cohabiting or married with women	12	2.2
Self-identified sex orientation		
Homosexual	470	86.7
Bisexual	53	9.8
Heterosexual/Uncertain	19	3.5
Exposure to HIV-related services in the last year		
Condom or lubricant distribution	368	67.9
Peer education	136	25.1
STD/HIV consultation	137	25.3
STD/HIV pamphlets	280	51.7
HIV voluntary counseling and testing (VCT)	279	51.5
Number of above types of HIV-related services utilizedin the last year		
0	79	14.6
1	112	20.7
2	122	22.5
3	110	20.3
4	81	14.9
5	38	7.0
STD history		
Self-reported having had STD-related symptoms in thelast year	54	10.0
Diagnosed as having had STD in the last year	4	0.7
Sexual behaviors in the last 6 months		
Number of male sex partners having hadanal intercourse with		
0	93	17.2
1	129	23.8
2–5	141	26.0
≥6	102	18.8
Can’t remember	77	14.2
Having had UAI with anymale partner		
Yes	175	32.3
No	367	67.7

Questions asked included knowledge and HBM-related cognitions on HPV and HPV vaccines (Tables 2 and 3). Six composite indicator variables were constructed in this study by counting the number of affirmative item responses reflecting the level of perceived susceptibility (ranged from 0 to 3), perceived severity (ranged from 0 to 3), perceived benefits (ranged from 0 to 5), perceived barriers (ranged from 0 to 5), perceived self-efficacy (ranged from 0 to 2) and perceived cue to action (ranged from 0 to 3).

**Table 2:** Frequency distributions of variables related to HPV-related perceptions (N = 542).

	N	%
Knowledge on HPV		
Whether males could be affected by HPV		
No	32	5.9
Yes*	257	47.4
Never heard of HPV/Don’t know	253	46.7
HPV was newly found in the last few years		
Yes/Uncertain	285	52.6
No*	257	47.4
HPV could be controlled by antibiotics		
Yes/Uncertain	409	75.5
No*	133	24.5
HPV is unlikely to be totally cured		
No/Uncertain	224	41.3
Yes*	318	58.7
HPV is hereditary		
Yes/Uncertain	266	49.1
No*	276	50.9
High mortality rate (>5%)		
Yes/Uncertain	362	66.8
No*	180	33.2
Number of appropriate response		
0	55	10.1
1	69	12.7
2	120	22.1
3	136	25.1
≥4	162	29.9
Perceived susceptibility of HPV infection		
Perceived chance of contracting HPV in the future		
Low/very low	363	67.0
Moderate	146	26.9
Very high/high	33	6.1
Perceived prevalence of HPV infection among MSM in Hong Kong		
≤10%	170	31.4
>10%	220	40.6
Uncertain	152	28.0
Perceived infectivity of HPV		
Low/very low	36	6.6
Moderate	210	38.7
Very high/high	296	54.6
Number of responses to the above 3 questions reflecting perceived susceptibility†		
0	164	30.3
1	221	40.8
≥2	157	29.0
Perceived severity of HPV infection		
Damages of HPV infection on physical health		
Low/very low	62	11.4
Moderate	200	36.9
Very high/high	280	51.7
Perceived chance of HPV infection causing genital warts		
Very low/Low/Moderate	197	36.3
Very high/high	345	63.7
Perceived chance of HPV infection causing penile/anal cancers		
Low/very low/uncertain	178	32.8
Moderate	167	30.9
Very high/high	197	36.3
Number of responses to the above 3 questions reflecting perceived severity of HPV infection†		
0	112	20.7
1	154	28.4
2	160	29.5
3	116	21.4

* Appropriate response.

† Number of affirmative responses (very high/high).

**Table 3:** Perceptions related to HPV vaccines and intention to take up HPV vaccines (N = 542).

	N	%
Knowledge on HPV vaccines		
Availability of effective HPV vaccines to men		
No/Don’t know	392	72.3
Yes*	150	27.7
Perceived price per shot (HK$: 1US$ = 7.8HK$)		
<800/>1500/Don’t know/Not available	461	85.1
800–1500*	81	14.9
Number of shots required		
1–2/4 or above/Don’t know/Not available	490	90.4
3*	52	9.6
Duration of protection		
1 year/2–5 years/5–10 years/Lifelong/Don’t know	521	96.1
10 years or above*	21	3.9
Age group best for HPV vaccination		
Above 30/All/Don’t know	492	90.8
Below 30*	50	9.2
Number of appropriate response to the above five questions on knowledge related to HPV		
0	293	54.1
1	170	31.4
2	60	11.1
≥3	19	3.5
Perceived benefits of HPV vaccines for preventing and treating diseases related to HPV		
Perceived efficacy in preventing genital warts		
Not very effective/not effective	58	10.7
Very effective/effective	367	67.7
Don’t know	117	21.6
Perceived efficacy in preventing HPV-induced cancers (penile and anal cancers)		
Not very effective/not effective	129	23.8
Very effective/effective	303	55.9
Don’t know	110	20.3
Perceived efficacy in preventing STD other than genital warts		
Not very effective/not effective	264	48.7
Very effective/effective	158	29.2
Don’t know	120	22.1
Perceived efficacy in treating genital warts		
Not very effective/not effective	187	34.5
Very effective/effective	207	38.2
Don’t know	148	27.3
Perceived efficacy in treating HPV-induced cancer (penile and anal cancers)		
Not very effective/not effective	281	51.8
Very effective/effective	93	17.2
Don’t know	168	31.0
Number of item responses to the above five questions reflecting perceived benefits of HPV vaccines†		
0	96	17.7
1	82	15.1
≥2	364	67.2
Perceived barriers to take up HPV vaccines		
HPV vaccination is expensive		
Totally disagree/disagree	151	27.9
Totally agree/agree	299	55.2
Don’t know	92	17.0
HPV vaccines could have side effects		
Totally disagree/disagree	180	33.2
Totally agree/agree	230	42.4
Don’t know	132	24.4
It is embarrassing to take up HPV vaccines		
Totally disagree/disagree	299	55.2
Totally agree/agree	224	41.3
Don’t know	19	3.5
It is troublesome to take up HPV vaccines		
Totally disagree/disagree	331	61.1
Totally agree/agree	168	31.0
Don’t know	43	7.9
Taking up HPV vaccine may be seen as a sign of promiscuity		
Totally disagree/disagree	340	62.7
Totally agree/agree	184	33.9
Don’t know	18	3.3
Number of item responses to the above five questions reflecting perceived barriers related to HPV†		
0	133	24.5
1	107	19.7
2	93	17.2
≥3	209	38.6
Perceived self-efficacy to take up HPV vaccines		
I am confident that I could take up HPV vaccines if I want to		
Disagree/Don’t know	60	11.1
Agree	482	88.9
I have full control on whether taking up HPV vaccines		
Disagree/Don’t know	25	4.6
Agree	517	95.4
Number of item responses to the above 2 questions reflecting perceived self-efficacy†		
0	17	3.1
1	51	9.4
2	474	87.5
Cue to action		
I have watched media reports promoting HPV vaccines among men		
No/Not sure	505	93.2
Yes	37	6.8
Doctor recommended me to take up HPV vaccines		
No/Not sure	528	97.4
Yes	14	2.6
Peer recommended me to take up HPV vaccines		
No/Not sure	524	96.7
Yes	18	3.3
Number of item responses to the above three questions reflecting cue to action received†		
0	488	90.0
1	43	7.9
≥2	11	2.1
Behavioral intention to take up HPV vaccines		
Intention to take up HPV vaccines within 6 months (unconditional)		
Definitely	21	3.9
Likely (Chance quite large)	87	16.1
Unlikely (Chance quite low)	259	47.8
Definitely not	93	17.2
Don’t know	82	15.1
Intention to take up HPV vaccines given efficacies in preventing genital warts and penile/anal cancer was 90% and 75%, conditional on the market price of $1000–2000 per shot for 3 shots within 6 months		
Definitely	29	5.4
Likely (Chance quite large)	129	23.8
Unlikely (Chance quite low)	225	41.5
Definitely not	159	29.3
Intention to take up HPV vaccines given efficacies and a discounted price ($500–1000 per shot and 3 shots within six months)		
Definitely	87	16.1
Likely (Chance quite large)	193	35.6
Unlikely (Chance quite low)	152	28.0
Definitely not	110	20.3
Intention to take up HPV vaccines given efficacies and three free shots in the next six months (given efficacies and free price)		
Definitely	308	56.8
Likely (Chance quite large)	121	22.3
Unlikely (Chance quite low)	67	12.4
Definitely not	46	8.5

* Appropriate response.

† Number of affirmative responses (very effective/effective, totally agree/agree, yes).

### Statistical Analysis

Odds ratios were firstly presented in the univariate analysis (ORu). A multiple stepwise regression model was then fit using the significant univariate background variables as candidates and multivariate odds ratios (ORm) were derived. Associations between the cognitive factors and the dependent variable (acceptability of HPV vaccination conditional on efficacies and market rate) were assessed, adjusting for those background variables that were found to be significant in the multivariate analysis and adjusted odds ratios (AOR) were derived. Corresponding 95% confidence intervals (CI) of odds ratios were presented. SPSS version 16.0 was used for data analysis, with p values of <.05 taken as statistically significant.

## Results

### Background Characteristics

About half of the participants (53.4%) were 30 years old or younger; 63.5% had attained colleges; 12.7% were cohabitating with a man; 86.7% considered himself as a homosexual person; 85.4% had utilized HIV prevention services; 10.0% had had some STD symptoms in the past year. The prevalence of UAI (last six months) was 32.3% and the prevalence of multiple male sex partnerships (last six months) was 44.8% (Table 1).

### Intention to Take Up HPV Vaccines in the Next Six Month (Acceptability)

The prevalence of acceptability was 20% (unconditional on efficacies and price), 29.2% (conditional on efficacies and the market price), 51.7% (conditional on efficacies and a halved discounted price) and 79.1% (conditional on efficacies and free price). Comparing participants of < = 26 and >26 years old, the differences in prevalence of acceptability of HPV vaccination under the aforementioned four scenarios were not found to be statistically significant. The prevalence of those who were < = 26 years old under the four scenarios was respectively 16.0%, 27.2%, 51.9% and 81.5%, whilst that of those who were >26 years old was respectively 18.9%, 30.0%, 51.6% and 78.2% (p>.05, chi-square test).

### Cognitions on HPV

The prevalence of appropriate response for the other individual knowledge items ranged from 24.5% to 58.7% (Table 2). There were hence common misconceptions that HPV would not affect men, that it could be controlled by using antibiotics, that it was a newly found virus, that it was totally curable, that it was hereditary and that it caused a mortality >5% (Table 2). Regarding perceived susceptibility, 40.6% of the participants perceived that the prevalence of HPV infection among MSM in Hong Kong was >10%; 54.6% believed that the infectivity of HPV was high or very high. In contrast, only 6.1% of the participants perceived a high or very high chance of contracting HPV in the future. Item responses reflecting perceived severity (Table 2) were: damages on physical health (51.7%), chance that HPV infection would cause genital warts (63.7%) and chance that HPV infection would cause penile or anal cancer (36.3%).

### Cognitions on HPV Vaccines

The prevalence on item responses corresponding to correct knowledge about HPV vaccines (availability, price, the 3-shot requirement, expected duration of protection, preferred age range for vaccination) was low and ranged only from 3.9% to 27.7% (Table 3). The prevalence of responses reflecting perceived benefits of HPV vaccines was: preventing genital warts (67.7%), preventing penile and anal cancers (55.9%), preventing STD other than genital warts (29.2%), treating genital warts (38.2%) and treating penile and anal cancers (17.2%). Concerning perceived barriers, the prevalence of agreement or strong agreement with the statements was: “HPV vaccines are expensive” (55.2%); “there are side effects” (42.4%); “it is embarrassing to take up HPV vaccines” (41.3%); “it is troublesome to take up HPV vaccines” (31.0%) and “HPV vaccination signifies promiscuity” (33.9%). The level of perceived self-efficacy to take up HPV vaccines was high (88.9% and 95.4%). The prevalence of responses reflecting exposure to cue to action was however, very low: media report (6.8%), doctor (2.6%) and peer (3.3%).

### Factors Associated with Acceptability of HPV Vaccines (Conditional on Specific efficacies for Disease Prevention and Market Price)

Two of the background variables that were listed in Table 4 were statistically significant: exposure to peer education (ORm = 1.55, 95% CI = 1.02–2.35) and UAI with any male sex partner (ORm = 0.47, 95% CI = 0.30–0.73). Adjusting for these two variables, the results showed that two knowledge variables were of marginal statistical significance (.05<p<.1): “don’t know whether men can be affected by HPV” (AOR = 2.36, 95% CI = 0.93–6.03) and perceived availability of effective HPV vaccines among men (AOR = 1.51, 95% CI = 0.99–2.29; Table 5). Two significant variables related to perceived severity of HPV included: perception that HPV had a high or very high chance of causing genital warts (AOR = 1.61, 95% CI = 1.06–2.43; reference group was “moderate/low/very low chance”) and perceived moderate or high/very high chances that HPV would cause penile/anal cancer (moderate chance: AOR = 3.55, 95% CI = 2.09–6.05; high/very high chance: AOR = 2.72, 95% CI = 1.61–4.60; reference group was “low/very low chance/uncertain”). Perceived efficacy in preventing STD other than genital warts (AOR = 1.69, 95% CI = 1.09–2.61) was the only item reflecting perceived benefits of HPV vaccines that was of statistical significance. Several factors on perceived barriers were significant: concerns on “expensive price” (AOR = 0.63, 95% CI = 0.41–0.97), “side effects” (AOR = 0.63, 95% CI = 0.41–0.98), and “promiscuity” (AOR = 0.38, 95% CI = 0.24–0.60) and some “don’t know” answers (e.g. “it is embarrassing to take up HPV vaccines”). All the three independent variables on perceived cue to action were significant: having obtained advices from the media (AOR = 2.49, 95% CI = 1.25–4.99), from doctors (AOR = 3.12, 95% CI = 1.05–9.30) and from his peers (AOR = 3.82, 95% CI = 1.43–10.21).

**Table 4:** Associations between background and intention to take up HPV vaccines (conditional on efficacies and $1000–2000 per shot for 3 shots to be taken within the future 6 months; N = 542).

Variables	Row %	OR_U_ (95%CI)	OR_M_ (95%CI)
Exposure to HIV-related services in the last year			
Peer education			
No	26.4	1	1
Yes	37.5	1.68 (1.11–2.53)*	1.55 (1.02–2.35)*
Sexual behaviors in the last 6 months			
UAI with any male partner			
No/no anal sex	34.1	1	1
Yes	18.9	0.45 (0.29–0.70)***	0.47 (0.30–0.73)**

* p<0.05;

** p<0.01;

*** p<0.001.

–: Univariately non-significant variables, not considered in the model.

ORu: univariate odds ratios.

ORm: multivariate OR, odds ratios obtained from stepwise multivariate logistic analysis using significant univariate variables as candidate variables.

95% CI: 95% confidence interval.

Variables that were not significant in the univariate analysis were not tabulated (age group, highest education attained, marital status, self-identified sex orientation, exposure to condom or lubricant distribution, STD/HIV consultation, STD/HIV pamphlets, VCT, STD history and number of male sex partners having anal intercourse with).

**Table 5:** Associations between factors related to HPV/HPV vaccine and the intention to take up HPV vaccines in the next six month (given efficacies and price of $1000–2000 per shot and 3 shots within in the next six months; N = 542).

	Row %	OR_U_ (95%CI)	AOR (95%CI)
Knowledge on HPV/HPV vaccine			
HPV was newly found in the last few years			
Yes/Uncertain	23.2	1	1
No	35.8	1.85 (1.27–2.69)**	1.78 (1.22–2.61)**
Whether males could be affected by HPV virus			
No	18.8	1	1
Yes	24.9	1.44 (0.57–3.65)	1.54 (0.60–3.95)
Don’t know/Never heard of HPV	34.8	2.31 (0.92–5.83)†	2.36 (0.93–6.03)†
Current availability of effective HPV vaccine to men			
No/Don’t know	26.3	1	1
Yes	36.7	1.62 (1.09–2.43)*	1.51 (0.99–2.29)†
Perceived susceptibility of HPV infection			
Perceived prevalence of HPV infection among MSM in Hong Kong			
≤10%	24.7	1	1
>10%	34.1	1.58 (1.01–2.46)*	1.50 (0.95–2.37)
Uncertain	27.0	1.13 (0.68–1.86)	1.22 (0.73–2.04)
Perceived severity of HPV infection			
Perceived chance of HPV infection causing genital warts			
Low/very low/Moderate	21.8	1	1
Very high/high	33.3	1.79 (1.19–2.69)**	1.61 (1.06–2.43)*
Perceived chance of HPV infection causing penile/anal cancer			
Low/very low/uncertain	14.0	1	1
Moderate	38.9	3.90 (2.31–6.59)***	3.55 (2.09–6.05)***
Very high/high	34.5	3.23 (1.93–5.40)***	2.72 (1.61–4.60)***
Perceived benefits of HPV vaccines for preventing and treating diseases related to HPV			
Perceived efficacy in preventing STD other than genital warts			
Not very effective/not effective	25.0	1	1
Very effective/effective	35.4	1.65 (1.07–2.53)*	1.69 (1.09–2.61)*
Don’t know	30.0	1.29 (0.80–2.08)	1.28 (0.79–2.09)
Perceived barriers to take up HPV vaccines			
HPV vaccination is expensive			
Totally disagree/disagree	38.4	1	1
Totally agree/agree	26.1	0.57 (0.37–0.86)**	0.63 (0.41–0.97)*
Don’t know	23.9	0.50 (0.28–0.90)*	0.54 (0.30–0.98)*
HPV vaccine could have side effects			
Totally disagree/disagree	37.8	1	1
Totally agree/agree	25.7	0.57 (0.37–0.87)**	0.63 (0.41–0.98)*
Don’t know	23.5	0.51 (0.31–0.84)**	0.52 (0.31–0.87)*
It is embarrassing to take up HPV vaccines			
Totally disagree/disagree	32.8	1	1
Totally agree/agree	22.3	0.59 (0.40–0.88)**	0.68 (0.45–1.03)
Don’t know	52.6	2.28 (0.90–5.79)	2.60 (1.00–6.74)*
Taking HPV vaccine can be seen as a sign of promiscuity			
Totally disagree/disagree	36.2	1	1
Totally agree/agree	15.8	0.33 (0.21–0.52)***	0.38 (0.24–0.60)***
Don’t know	33.3	0.88 (0.32–2.41)	0.97 (0.35–2.67)
Cue to action to take up HPV vaccines			
I have watched media reports promoting HPV vaccines for men			
No/Not sure	27.7	1	1
Yes	48.6	2.47 (1.26–4.84)**	2.49 (1.25–4.99)**
Doctor have recommended me to take up HPV vaccines			
No/Not sure	28.4	1	1
Yes	57.1	3.36 (1.15–9.85)*	3.12 (1.05–9.30)*
My peers have recommended me to take up HPV vaccines			
No/Not sure	28.1	1	1
Yes	61.1	4.03 (1.53–10.60)**	3.82 (1.43–10.21)**

* p<0.05;

** p<0.01;

*** p<0.001,

† 0.05<p<0.1;

Univariately non-significant variables were not listed in this table.

ORu: univariate odds ratios.

AOR: adjusted OR, odds ratios adjusting for all multivariately significant background variables listed in Table 1, including peer education and UAI with any male partner.

95% CI: 95% confidence interval.

Furthermore, the adjusted analysis showed that four of the six HBM-related composite indicator variables (see Measures) on perceived susceptibility (AOR = 1.85 to 2.07, p<.05), perceived barriers (AOR = 0.59, p<.05), perceived cue to action (AOR = 25.99, p<.05), and perceived severity (AOR = 1.89 to 1.94, p<.05) were of statistical significance (Table 6). In addition, we fit a multiple logistic regression model containing all the factors that were listed in Table 6, adjusted for the same significant background variables. Similar results were obtained except that the variable of perceived severity became statistically non-significant, due to its associations with other variables included into the model (data not tabulated).

**Table 6:** Associations between composite cognitive indicator variables) and intention to take up HPV vaccines in the next six months (given efficacies and the market price of $1000–2000 per shot and three shots be taken within six months; N = 542).

	Row %	OR_U_ (95%CI)	AOR(95%CI)
Perceived susceptibility of HPV (number of items with affirmative responses)			
0	18.9	1	1
1	32.1	2.03 (1.25–3.29)**	1.85 (1.13–3.02)*
≥2	35.7	2.38 (1.43–3.96)**	2.07 (1.23–3.48)**
Perceived severity of HPV (number of items with affirmative responses)			
0	17.0	1	1
1	31.2	2.22 (1.22–4.04)**	1.92 (1.05–3.54)*
2	32.5	2.36 (1.30–4.27)**	1.89 (1.02–3.49)*
3	33.6	2.48 (1.33–4.64)**	1.94 (1.02–3.70)*
Perceived barriers to take up HPV vaccines (number of items with affirmative responses)			
0	36.1	1	1
1	29.9	0.76 (0.44–1.30)	0.79 (0.46–1.37)
2	34.4	0.93 (0.53–1.62)	1.00 (0.57–1.75)
≥3	22.0	0.50 (0.31–0.81)**	0.59 (0.36–0.96)*
Cue to action to take up HPV vaccines (number of items with affirmative responses)			
0	27.5	1	1
1	32.6	1.28 (0.65–2.49)	1.22 (0.62–2.40)
≥2	90.9	26.42 (3.35–208.36)**	25.99 (3.24–208.46)**

* p<0.05;

** p<0.01.

–: Univariately non-significant variables, not considered in the model.

ORu: univariate odds ratios.

AOR: adjusted OR: odds ratios adjusting for all multivariately significant background variables listed in Table 1, including peer education and UAI with any male partner.

95% CI: 95% confidence interval.

Composite variables that were not significant in the univariate analysis were not tabulated (number of appropriate response related to knowledge on HPV vaccines, perceived benefits of HPV vaccines preventing and treating diseases related to HPV and perceived self-efficacy on taking up HPV vaccines).

## Discussion

Like their counterparts in other countries (e.g. [39]), the sampled MSM were at high risk of contracting HPV as many of them had had STD symptoms, UAI with men and multiple male sex partners. Such observations corroborated with the results obtained from a number of local studies (e.g. [40]). It was however, unexpected that those who had had UAI were significantly less likely than others to find HPV vaccines acceptable. It is plausible that the motivation to use condoms correlates closely with that of taking up HPV vaccines as both measures are means of HIV/STD prevention. Those who care less about HIV/STD infection would neither use condoms during anal intercourse nor be willing to take up HPV vaccination. A positive association between UAI and lower acceptability of HPV vaccination would then be resulted. Therefore, we cannot expect MSM of higher risk of HIV/STD transmission would naturally become more willing to take up HPV vaccination because of their high risk status and their stronger wish to protect themselves. Instead, interventions to promote HPV vaccination targeting high risk MSM are required to persuade them to adopt this and other preventive measures.

It is known that the cost of the HPV vaccine is a strong determinant of HPV vaccination among females [41]. No study has studied the association between price sensitivity of HPV vaccines and acceptability of HPV vaccines among MSM. Our results showed that acceptability of HPV vaccines varied from about 30% to about 50% if the prices were halved to almost 80% if free vaccines were made available. Our prevalence of acceptablity of HPV vaccines at market price was lower than those reported in the U.S. which did not condition on price, but was comparable to the Australian data which conditioned on price. Our acceptability data conditioned on free vaccines was comparable to or higher than those reported in the U.S. studies. It is important to point out that five of the six existing reports on acceptability of HPV vaccines among MSM had not mentioned price of the vaccines [27], [28], [29], [30], [31]. The results of such studies were difficult to interpret. A number of acceptability studies on new public health initiatives have found significant differences between conditional and unconditional acceptability [42]. Conditional measures are hence required for acceptability studies. It is also important to lower the cost of HPV vaccination. For public health reasons, some governments have subsidized various types of vaccination among various high risk groups [43]. Previous studies have already proved that HPV vaccination among young MSM is cost effective [10], consideration should hence be made to provide free or low-cost HPV vaccination to high risk young MSM, such as male sex workers, which may maximize the benefit.

It was found that MSM did not know enough about HPV, as close to half of them possessed misconceptions about HPV (HPV would not affect men; HPV could be controlled by antibiotics; HPV was a newly found virus; HPV is totally curable and HPV is hereditary) and 72.3% of them did not know about availability of HPV vaccination for men. Such knowledge may potentially affect perceived needs to adopt preventive behaviors as it is associated with perceived susceptibility and perceived severity. To promote the newly available HPV vaccines among MSM, we hence need to increase their knowledge about HPV and HPV vaccines (e.g. diseases caused, availability to men, duration of protection and effectiveness), as we found that the knowledge levels were low and some of the HPV-related knowledge variables were significantly associated with acceptability of HPV vaccination.

Four HBM-related composite indicator variables (perceived barriers, perceived susceptibility, perceived severity and cue to action) were significantly associated with acceptability of HPV vaccines. It is important to remove perceived barriers. In this study, cognitive and psychological barriers (perceived side effects, potential embarrassment and worry about the label of promiscuity) instead of logistical barriers (e.g. troublesome to take up the vaccines) were of statistical significance. It is known that worrying about side-effects is a common determinant of acceptability of new vaccines [44]. Furthermore, genital warts and genital/anal cancers may be associated with social stigma, which is prevalent among MSM [45]. Those taking up HPV vaccines may be labeled as being promiscuous [46]. To minimize stigma resulted from HPV vaccination, programs promoting HPV vaccines should be conducted by peer MSM and at NGO that serve MSM; the MSM communities should always be involved to provide advice to the design of such programs. Good doctor-patient communication is also required to reduce embarrassment [47]. The vaccination should also be backed up by MSM-friendly clinics as previous studies have reported discrimination against MSM in health care settings [48]. Therefore, clinical, financial and psycho-social factors all need to be carefully considered when designing effective programs promoting HPV vaccination among MSM.

Perceived susceptibility was another statistically significant factor. Though high percentages of the participants perceived high infectivity of HPV and high prevalence of HPV among local MSM, only about 6% of them perceived a high or very high chance in contracting HPV in the future. This phenomenon of having high perceived risk for others but low perceived risk for oneself in contracting HIV/STD has been reported in previous studies [49]. It is important to increase risk perception of HPV among MSM. Dissemination of data about the high prevalence of HPV among MSM should be a good starting point. Both perceived severity of genital warts and penile/anal cancer were significant factors. Promotion campaigns should emphasize on both diseases. It is uncertain whether focusing only on the cancer factor would suffice. If such is true, hindrance of HPV vaccination due to stigma associated with genital warts could be reduced. Further studies are warranted.

Cue to action is strongly associated with vaccination behaviors [50]. In the Health Belief Model, cue to action is an important determinant of the intention to take up a health-related behavior such as safer sex. In this study, all three types of cues to action (media, doctors, and peers) were significant. Media campaigns have been used effectively to promote HPV among young women [51]. Gay websites may be an important media accessing MSM as the prevalence of internet use among MSM is very high [52]. Involvement of health professionals is always important [53]. Peer education and testimonials may be considered.

Perceived benefit was not significantly associated with acceptability of HPV vaccines. The participants were briefed about the vaccines’ efficacies before they answered the questions on acceptability of HPV vaccines; questions on perceived benefits were asked after the questions on acceptability. The sequence may have reduced the strength of this association. One single item on perceived benefit was significant (“Perceived efficacy in preventing STDs other than genital warts”). Genital warts increase the risk of contracting other types of STD [54]. Promotion campaigns should explain such relationships to MSM. Perceived self-efficacy was also non-significant. This may be explained by the small variation in item responses, as about 90% of the participants perceived a high level of self-efficacy on HPV vaccination.

Promotion of HPV vaccines is warranted and the social context seems favorable for such campaigns. MSM in China, including Hong Kong, tend to be young and well educated. In general, younger age and higher education levels are associated with adoption of preventive behaviors [55]. As recommended by international and local health authorities, HPV vaccines work well for younger men, an important message to be disseminated to MSM. There is a good chance to integrate promotion of HPV vaccination with existing HIV prevention services. About half of the participants had taken up HIV voluntary Counseling and Testing (VCT) and regular VCT is recommended. It is potentially useful to include HPV vaccination as part of the VCT services offered by the government and NGO. The integration may be effective as VCT users may already been motivated to take up HIV protective measures and should be more ready to take up HPV vaccines, as compared to their counterparts who are unwilling to take up VCT. The integration of HPV and HPV vaccination may even attract more MSM to take up VCT as HPV vaccination becomes an added value to existing HIV VCT. Stigma may be less an issue in the context of having VCT provided by peer educators. It is suggested that HIV workers need to be trained about HPV and HPV vaccination. Pilot studies to evaluate effectiveness of promoting HPV vaccination in settings of HIV VCT and other HIV-related service are greatly warranted.

Our sampling method has some limitations. The sample may be subjected to selection bias since no sampling frame existed and convenience sampling involving multiple sources was conducted. We did not recruit participants from the Internet though about half of the local MSM have recruited male sex partners from this source [56]. In addition, we did not systematically collect information about the gay bars and saunas that accepted or refused our invitation to participate the study. The response rate was only about 50% though it is comparable to that of some similar studies [57]. Several other limitations should also be brought to readers’ attention. First, this is a cross-sectional study and causal relationship cannot be established. Second, we asked about behavioral intention rather than actual behaviors. It is expected that the prevalence of HPV vaccination approached zero as HPV vaccines have only been newly available to males. Behavioral intention, an important construct of the Theory of Planned Behaviors [58], is strongly predictive of health-related behaviors. Third, social desirability may lead to over-reporting of acceptability. The question items were constructed for this study as no validated scale was available. Lastly, the sequence of asking the four conditions of acceptability of HIV vaccination (overall, efficacies and market rate, efficacies and discounted rate, efficacies and free vaccination) may potentially affect the responses to these items. However, since our associated factors were based on acceptability of HPV vaccination at the market rate (asked in the second place), we believe that the effect of the sequence of questions on the reported associations should be acceptable.

### Conclusion

In sum, HPV vaccination among MSM is an important public health initiative and promotion is warranted. Such campaigns need to focus on cognitions and knowledge related to HPV and HPV vaccines. To recapitulate, the studies have important implications for designing HPV vaccination programs. Such programs should start with media campaigns promoting knowledge about HPV and HPV vaccination among MSM. The Health Belief Model can be used as a framework to guide design of such programs as all except one of the constructs (self-efficacy) were found to be significantly associated with acceptability of HPV vaccination in this study. Enhanced risk perception would increase perceived susceptibility of HPV infection. Increased perceived severity can be achieved by emphasizing the causation between HPV and both penile/anal cancers and genital warts. Such programs need to remove both financial and psychological barriers. Discounted low-cost or free HPV vaccination schemes should be provided to high risk MSM such as male sex workers and HIV-infected MSM. Psychological barriers also need to be removed, possibly by collaborating with MSM-friendly NGO and empathetic clinicians. Media campaigns tailored to accessing MSM and new approaches involving health professionals and peers should be used as cues to actions to regularly remind MSM to take up HPV vaccination. In social marketing, marketing mix includes manipulation of product, price, place and promotion to facilitate behavioral change [59]. In our case, the key product is the HPV vaccination program. The price factors include aforementioned reduction of the cost (both psychological and financial cost) and increase in incentive of vaccination by emphasizing on its efficacy in preventing HPV, which is prevalent and results in both cancer and genital warts. The place factor refers to potential vaccination program which can be offered at NGO venues or gay-friendly clinics. The promotion factor includes promotion through social media, gay-webpage, STD clinics, gay venues and NGO venues. We can therefore use the social marketing approach, which has shown to be effective in changing many health-related behaviors [60] including those related to HIV [61], [62], to design HPV vaccination promotion programs. No such program has however been reported. At the policy level, long-term plans to integrate STD (including HPV infection) and HIV prevention should be considered. Our results show that about 30% of the MSM population would take up the vaccines at the current market price. The acceptability of HPV vaccination among MSM is expected to increase substantially after the implementation of some health promotion programs. Pilot implementation trials of promotion of HPV vaccination are greatly warranted.
